# Active components of *Patrinia scabiosifolia* and mechanisms of action against methicillin-resistant *Staphylococcus Epidermidis*

**DOI:** 10.3389/fcimb.2026.1813767

**Published:** 2026-07-15

**Authors:** Qiantonghan Luo, Wenqiang Cui, Meng Ni, Yuqi Yang, Yonghui Zhou, Lili An, Xin Liu

**Affiliations:** 1 Guizhou University of Traditional Chinese Medicine, Guiyang, China; 2 The Research Center for Computer-aided Drug Discovery, Institute of Biomedicine and Biotechnology, The Shenzhen Institute of Advanced Technology, Chinese Academy of Sciences, Shenzhen, China; 3 University of Chinese Academy of Sciences, Beijing, China; 4 The First Affiliated Hospital of Guizhou University of Traditional Chinese Medicine, Guiyang, China

**Keywords:** Methicillin-resistan *Staphylococcus epidermidis*, Bloodstream infections, *Patrinia scabiosifolia*, Arginine deiminase, Hexadecanal

## Abstract

**Introduction:**

*Staphylococcus epidermidis* is a significant cause of hospital bloodstream infections, often transmitted through medical devices. Methicillin-resistant *Staphylococcus epidermidis* (MRSE) exhibits multidrug resistance, challenging clinical treatment. Current options are limited, with vancomycin as the primary choice despite its negative effects. Traditional Chinese Medicine (TCM) is a valuable resource for novel antibiotics; Our research showed *Patrinia scabiosifolia* has potent anti-MRSE activity. TCM’s efficacy stems from active components, but active components and mechanisms of *Patrinia scabiosifolia* are unclear. Arginine deiminase (AD) directly promotes bacterial growth by participating in protein synthesis, energy metabolism, and intermediate product formation, serving as a key substance for bacterial adaptation and survival. Our preliminary results demonstrated that *Patrinia scabiosifolia* can effectively inhibit the expression of AD in MRSE. Thus, we propose that AD serves as the antibacterial target of *Patrinia scabiosifolia* against MRSE. TCM's efficacy stems from active components, but active components and mechanisms of *Patrinia scabiosifolia* are unclear. Consequently, it has emerged as a potential target for combating bacterial and drug-resistant infections. Therefore, this study is designed to screen active components of *Patrinia scabiosifolia* targeting AD and further investigate the underlying antibacterial mechanisms.

**Methods:**

To validate the hypothesis, we constructed an *arcA* deletion strain (*ΔarcA*), in which the *arcA* gene encoding the AD protein was deleted via homologous recombination. Antimicrobial experiments *in vitro* and *in vivo* confirmed it as one target of *Patrinia scabiosifolia*. Furthermore, the active components of *Patrinia scabiosifolia *with anti-MRSE activity were screened by molecular docking and molecular dynamics (MD) simulations, using AD as the molecular target. In addition, the *in vitro* and *in vivo* antibacterial activities of the active components from *Patrinia scabiosifolia* were investigated. At last, the regulatory effect of the active components of *Patrinia scabiosifolia* on AD was determined by measuring the expression of the metabolite *arcA* gene and ornithine; Meanwhile, the binding effect of the active components of *Patrinia scabiosifolia* on AD was confirmed via determining the binding of AD to these components by microscale thermophoresis (MST), as well as identifying the binding sites through molecular simulation. Finally, nitric oxide (NO) and catalase (CAT) were determined, which further illustrates the antibacterial mechanism of the active components of *Patrinia scabiosifolia* targeting AD.

**Results:**

This study successfully constructed an MRSE arcA-deletion strain (*ΔarcA*), whose *arcA* gene encodes the AD protein, confirming that the deletion strain exhibited no growth defects. The minimum inhibitory concentration (MIC) of *Patrinia scabiosifolia* against MRSE was 5 mg/mL, whereas its MIC against *ΔarcA* was 10 mg/mL. Through MD simulations, 21 active components with anti-MRSE activity were screened from *Patrinia scabiosifolia*, among which hexadecanal demonstrated high antibacterial activity—showing a MIC of 7.81 μg/mL and a minimum bactericidal concentration (MBC) of 125 μg/mL against MRSE, and a MIC of 31.25 μg/mL against *ΔarcA* with no detectable MBC. Measurements of *arcA* gene expression and ornithine levels in MRSE treated with or without hexadecanal indicated that hexadecanal significantly downregulated *arcA* gene expression and reduced ornithine levels. MST and molecular simulation experiments further confirmed that hexadecanal binds to AD with high affinity, and identified Phe148 as its key binding site. In-depth studies revealed that hexadecanal targets AD, leading to marked reductions in NO and CAT levels.

**Conclusions:**

This study elucidates that hexadecanal serves as the active components of *Patrinia scabiosifolia* targeting AD, and clarifies its antibacterial mechanism via binding to AD to regulate AD function and subsequently initiates oxidative stress.

## 1 Introduction

*Staphylococcus epidermidis* (*S. epidermidis*), a notorious opportunistic pathogen, has emerged as one of the most significant causes of hospital-acquired infections. When the body undergoes implant-related treatment, this bacterium may breach host defenses or spread to distant sites by colonizing indwelling venous catheters, artificial heart valves, artificial joints, and other medical grafts. It can trigger serious conditions like bacterial endocarditis, neonatal septicemia, and periprosthetic infections (Leistikow et al., 2024; Paramanya et al., 2024). Among these, bloodstream infection represents a particularly grave threat. Any discussion about *S. epidermidis* as a pathogen is invariably qualified by the comment that it lacks the diverse array of virulence factors found in *Staphylococcus aureus* (Burke et al., 2024). However, *S. epidermidis* excels at evading the host immune system, leading to persistent, long-term infections, and readily acquires resistance to antibiotics (Månsson et al., 2021; Shaikh et al., 2025). The treatment of chronic infections is complicated by the intrinsic resistance of bacteria, resulting in a substantial burden for public health systems. Moreover, the drug resistance rate of *S. epidermidis* has been increasing year by year and even the phenomenon of multiple drug resistance has appeared (Kellgren et al., 2024). Among these the prevalence of methicillin-resistant *Staphylococcus epidermidis* (MRSE) has ranged from 34 to 79% (Goss et al., 2024; Guo et al., 2024). The MRSE showed a broad drug resistance spectrum including β-lactam, quinolone, macrolide, aminoglycosides, and so on (Liu et al., 2023). At present, vancomycin has a good effect on systemic infection caused by MRSE. However, its toxicity, low tissue permeability, drug resistance, and weak inhibition effect of biofifilm have limited its clinical application (Ramos et al., 2023; Huang et al., 2025). Therefore, finding effective drugs or alternative methods to treat MRSE infections is urgently required.

Traditional Chinese medicine (TCM) is believed to be an important source for the screening and development of new anti-durg resistant bacterial (Liu et al., 2022a). Moreover, clinical observations and laboratory studies indicate that TCM holds remarkable potential for treating drug-resistant bacterial (Liu et al., 2023); Furthermore, our previous research demonstrated that *Patrinia scabiosifolia* exhibits potent antibacterial activity against MRSE (Liu et al., 2023). Therefore, *Patrinia scabiosifolia* emerges considered a promising candidate for treating MRSE infections.

The complexity of most TCM mixtures in terms of chemical composition could hinder both their standardization and wider applications. Artemisinin stands as the first innovatively developed agent from TCM, it is the first monomeric TCM-derived drug enlisted in the World Health Organization (WHO) International Pharmacopoeia (Li, 2012). Therefore, research into the material basis of TCM holds the key to achieving modernization of TCM. However, current studies are largely confined to analyzing the chemical components of *Patrinia scabiosifolia*, with scant research on bioactive compound for its pharmacological effects, especially the antibacterial effects (Al-Shuhaib and Al-Shuhaib, 2024). On this account, it is necessary to study the active ingredients of *Patrinia scabiosifolia.*

**Figure 1:**
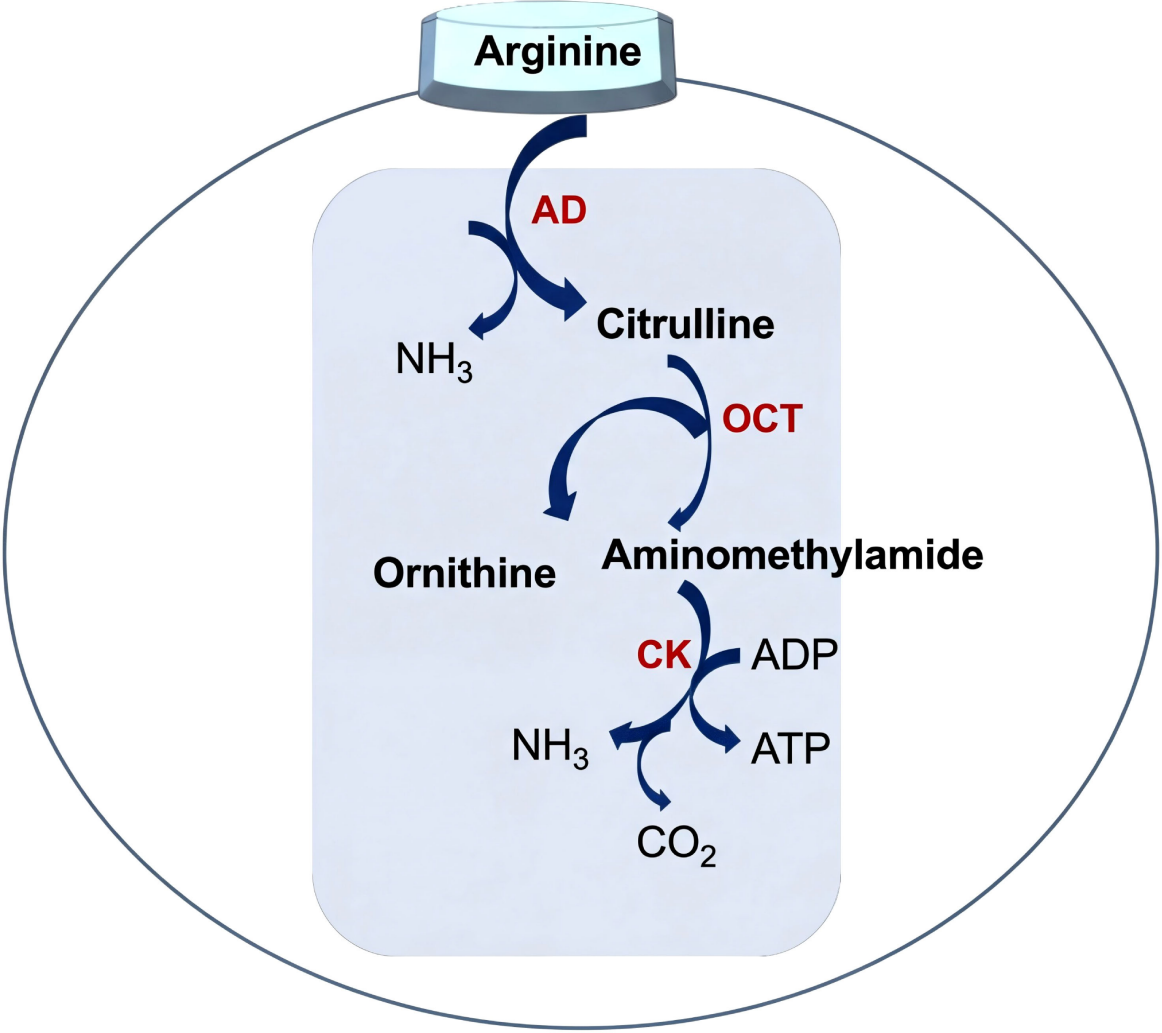
Arginine deiminase pathway. This diagram comprehensively illustrates the classical arginine catabolic pathway in microorganisms, which is sequentially catalyzed by three core enzymes (AD, OCT, CK). This pathway converts extracellularly ingested arginine into ornithine while producing ATP, ammonia (NH_3_), and carbon dioxide (CO_2_), thereby providing energy for the cell and contributing to intracellular acid-base homeostasis regulation.

Arginine metabolism is considered to be closely associated with bacterial virulence and drug resistance, and has become a promising target for the development of novel antibacterial agents (Sullivan et al., 2021). The arginine deiminase pathway is one of the predominant metabolic routes for arginine and widely distributed in diverse prokaryotes. This pathway consists of three key enzymes: arginine deiminase (AD), ornithine carbamoyltransferase (OCT), and carbamate kinase (CK) (**Figure 1**) (Sullivan et al., 2021).

As the first catalytic enzyme in the arginine deiminase pathway, AD has been identified as a target protein against methicillin-resistant *Staphylococcus aureus* (MRSA) (Moreno et al., 2022). Meanwhile, AD is absent from the human genome, which helps avoid toxic side effects of candidate drugs on humans and mammals (Freiberg et al., 2024). Accordingly, AD has emerged as a novel target for antibacterial drug development. Our previous proteomic studies revealed that AD in MRSE is significantly downregulated under *Patrinia scabiosifolia* (Liu et al., 2022b). In consequence, we believe that Patrinia scabiosifolia exerts antibacterial effects against MRSE through AD potentially. Therefore, this study intends to screen active constituents targeting AD and further elucidate their antibacterial mechanisms mediated by AD inhibition.

In this study, an MRSE *ΔarcA* mutant was generated via homologous recombination, and in vitro antimicrobial activity assay that AD is the target of *Patrinia scabiosifolia* against MRSE. we integrated molecular docking and molecular dynamics (MD) simulations to identify bioactive constituents of *Patrinia scabiosifolia* with potent anti-MRSE activity by targeting AD. The antibacterial efficacy of the bioactive compound is further rigorously validated through comprehensive *in vitro* and *in vivo* assays. Mechanistic investigations revealed that the bioactive compound exerts anti-MRSE effects by binding to and modulating the function of AD. Collectively, our findings demonstrate that the bioactive compound from *Patrinia scabiosifolia* directly targets AD to inhibit MRSE, providing molecular insights and a strategic framework for developing next-generation therapeutics against MRSE infections.

## 2 Materials and methods

### 2.1 Strains

In this study, the bacterial strain *S. epidermidis* ATCC12228 was obtained from American Type Culture Collection. The MRSE was previously induced under methicillin from *S. epidermidis* ATCC12228 and preserved in our lab. The MRSE is cultured at 37°C. The strain has been identified by routine laboratory methods and stored in 20% (v/v) glycerol at −80°C.

### 2.2 Constructions of arcA knockout bacteria

The *arcA* knockout strain of MRSE was generated using the plasmid pKOR1. In brief, the upstream and downstream fragments of *arcA* were amplified from the genomic DNA of MRSE. The PCR products were subsequently cloned into pKOR1 utilizing T4 DNA Ligase (Takara), resulting in the construction of a plasmid designated as pKOR1-*arcA*. Following modification with RN4220, this plasmid was transduced into MRSE via phage transduction. The knockout cells were identified on TSA plates supplemented with 10 μg/mL−1 chloramphenicol. Genome editing was confirmed through PCR analysis using extracted genomic DNA and further validated by DNA sequencing. Additionally, the plasmid within the mutant strain was eliminated according to a previously established protocol (Wencker et al., 2024).

### 2.3 Determination of minimum inhibitory concentration

The broth microdilution method was employed to determine the minimal inhibitory concentration (MIC) of *Patrinia scabiosifolia*, accordance with the outlined by the Clinical and Laboratory Standards Institute (Weinstein, 2021). The MRSE and *ΔarcA* treated with *Patrinia scabiosifolia* were incubated at 37˚C for 18h. The MIC was considered as the lowest concentration of *Patrinia scabiosifolia* with no visible microbial growth after 18h of incubation. This experiment was repeated three times.

### 2.4 Computational discovery of AD-targeting compounds

#### 2.4.1 Protein preparation

The arginine deiminase (AD) structure was obtained from UniProt (Q8CMW1) and predicted using AlphaFold2 (Jumper et al., 2021). Model confidence was assessed using the predicted Local Distance Difference Test (pLDDT) scores provided by AlphaFold, with 81.8% of residues classified as very high confidence and an average pLDDT score of 93.81, indicating a highly reliable structural model. Protein preparation was carried out in the Schrödinger Suite using the OPLS_2005 force field. Hydrogen atoms were added and protonation states at physiological pH (7.0) were optimized with PROPKA to refine the hydrogen-bonding network (Reis et al., 2024). The resulting structures were subjected to constrained energy minimization with a heavy-atom convergence threshold of 0.3 Å. The 3D structures of gallic acid was retrieved from Pubchem daatabase (https://pubchem.ncbi.nlm.nih.gov/).

#### 2.4.2 Ligand preparation

Active constituents of *Patrinia scabiosifolia* were collected from the Traditional Chinese Medicine Systems Pharmacology Database (TCMSP). (https://www.tcmsp-e.com/). TCMSP provides ADME-related pharmacokinetic descriptors (Liu et al., 2022c) based on established criteria (Huang et al., 2023). Compounds with oral bioavailability (OB) ≥ 30% and drug-likeness (DL) ≥ 0.18 were selected for further analysis. Ligand geometry optimization was performed using the LigPrep module using OPLS_2005 force field (Fan et al., 2020). Epik was used to generate relevant ionization and tautomeric states employing the Hammett–Taft formalism (Kersten et al., 2024).

#### 2.4.3 Virtual screening

Structure-based virtual screening was conducted using the Glide module in the Schrödinger Suite (Wang et al., 2024). The docking grid was generated around the active site of AD based on the ligand-bound arginine deiminase crystal structure from Pseudomonas aeruginosa (PDB ID: 2A9G). A cubic grid box (20 Å radius) centered on the co-crystallized ligand was used for all docking calculations. Standard Precision (SP) Glide docking was performed, and the top-ranked pose for each ligand was selected for subsequent molecular dynamics (MD) simulations.

### 2.5 Molecular dynamics simulations

MD simulations were carried out using Desmond. Ligand parameters were assigned using the OPLS_2005 force field (Chakravorty et al., 2024). Ach protein–ligand complex was solvated in an orthorhombic SPC water box with 10 Å padding in all directions. Counterions (Na^+^/Cl^-^) were added to neutralize the system, and physiological ionic strength (0.15 M NaCl) was maintained. The M-SHAKE algorithm was applied to constrain bonds involving hydrogen atoms. Equilibration was performed for 200 ps under NPT conditions (310 K, 1 bar). Production simulations were run for 200 ns. Trajectory analyses, including RMSD, RMSF, and binding interactions, were performed using Maestro and PyMOL (Bramucci et al., 2012).

### 2.6 Determination of minimum inhibitory concentration

*In vitro*, the broth microdilution method was employed to determine the MIC of the bioactive compound in *Patrinia scabiosifolia*, as outlined by the Clinical and Laboratory Standards Institute (Weinstein, 2021). The MRSE and *ΔarcA* treated with the bioactive compound in *Patrinia scabiosifolia* were incubated at 37˚C for 18h. The MIC was considered as the lowest concentration of the bioactive compound in *Patrinia scabiosifolia* with no visible microbial growth after 18h of incubation. The assay was performed in triplicates.

### 2.7 Assessment of minimum bactericidal concentration

The broth microdilution method was employed to determine the MIC of hexadecanal, as outlined by the Clinical and Laboratory Standards Institute (Alrashidi et al., 2021). The MRSE and *ΔarcA* treated with hexadecanal were incubated at 37˚C for 18h. Subsequently, transfer 10 μL of bacterial suspension from wells with MIC and higher concentrations, spread evenly on TSA agar plates, and incubate at 37˚C for 18 hours before performing colony counting. The MBC was defined as the lowest concentration of an antimicrobial agent capable of inactivating > 99.99% of the bacterial population (< 10 CFU/mL). The assay was performed in triplicates.

### 2.8 Establishing the time-kill curve for hexadecanal

The time-kill curve is used to dynamically evaluate the bactericidal kinetics of antimicrobial agents against bacteria (Alrashidi et al., 2021). First, prepare MRSE bacterial suspension in the logarithmic growth phase (approximately 1.0×10^6^ CFU/mL). Dilute hexadecanal to various concentrations including 0 MIC, 1/2 MIC, MIC, 2 MIC, 4 MIC, and 16 MIC. After inoculation, collect 10 μL samples at time points of 0, 1, 2, 4, 8, 12, and 24 hours, perform gradient dilution, and plate them. After 24 hours of incubation, count the colonies and calculate Log_10_ CFU/mL and the bactericidal rate. The assay was performed in triplicates.

### 2.9 Treatment of hexadecanal against MRSE-induced bloodstream infection

*In vivo*, Male c57bl/6j mice between six and eight-weeks-old were used in this study. 120 mg/k cyclophosphamide (CP) treatment regimen was used to induce immunosuppressive model. Furthermore, MRSE -induced Bloodstream Infection models (MRSE and *ΔarcA*)were established according to the procedures described in previous articles (Liu et al., 2023). The mice were randomly separated into eight groups (n=6), including immunosuppressive group, Wilde-type (WT) bloodstream infection group, *ΔarcA* bloodstream infection group, hexadecanal treatment for WT bloodstream infection group, hexadecanal treatment for *ΔarcA* bloodstream infection group, DMSO for WT bloodstream infection group, DMSO for *ΔarcA* bloodstream infection group and blank group. Hexadecanal (2.5 μg/g) was administered by intraperitoneal injection at seven days before infection in hexadecanal treatment for WT bloodstream infection group, hexadecanal treatment for *ΔarcA* bloodstream infection group. Finally, the blood of the mice in each group was obtained and, the number of clones were counted The above mouse experiment was approved by the University Committee on Use and Care of Animals at the Guizhou University of Traditional Chinese Medicine and done as Laboratory Animal – Guideline for Ethical Review of Animal Welfare (Ethics approval number: 20230147).

### 2.10 Quantitative Real-Time PCR (qRT-PCR) analysis

Quantitative RT-PCR was employed to the determination of *arcA* expression in MRSE with or without Hexadecanal, as the previous experimentina (Liu et al., 2023). qRT-PCR was carried out using SYBR Premix Ex Taq on StepOne Real-Time PCR System. The reaction conditions were 94 °C for 10 min followed by 40 cycles of amplification at 94°C for 15s and 60 °C for 60s. The assays were repeated 3 times. Each containing three replicates for *arcA* and the relative fold changes were calculated using the 2−ΔΔCt method.

### 2.11 Determination of ornithine content

High-Performance Liquid Chromatography (HPLC) was used to determine ornithine content. The MRSE and *ΔarcA* treated with hexadecanal were incubated at 37˚C for 18h. The chromatographic conditions are as follows: chromatographic column: Information-HILICZ (2.7 μm, 3.0 × 100 mm); column temperature maintained at 35°C; mobile phase consisting of Phase A (water, pH=3) and Phase B (premixed 90% acetonitrile - 10% water, pH=3); flow rate set at 0.3 mL/min; injection volume of 1 μL; detection wavelength fixed at 254 nm. The details are listed in **Supplementary Table 1S** of the supplementary materials. In addition, the mass spectrometry parameters are listed in **Supplementary Table 2S** and **Supplementary Table 3S**.

### 2.12 Expression and purification of AD and AD’s site-directed mutant protein

Expression and purification of AD and AD’s site-directed mutant protein were done as Yuan (Yuan et al., 2023). The AD protein and its site-directed mutant (Phe 148 to Lys) were produced using the pET30a vector. The *arcA* gene, which encodes the AD protein, was synthesized by Integrated DNA Technologies based on its sequence from *S. epidermidis* ATCC12228. The PCR product was subsequently ligated into the pET30a vector via In-Fusion cloning. Recombinant plasmids were transformed into DH5α competent cells, followed by collection of these plasmids for transformation into BL21 competent cells. Isopropyl-β-D-thiogalactopyranoside was added to a final concentration of 1mM to induce expression of both the AD protein and its site-directed mutant in BL21 cells. The expressed proteins were purified using a Ni-NTA agarose column and an AKTA Pure Protein Purification System (GE, USA) from harvested bacterial suspensions. Finally, the quality and quantity of purified recombinant AD protein and its site-directed mutant were analyzed via 10% SDS-PAGE gel electrophoresis.

### 2.13 Determination of microscale thermophoresis

MST was used to quantify the hexadecanal binding affinities of AD and AD’s site-directed mutant protein as previously reported (Moldovean-Cioroianu, 2024). A volume of 100 mL (10 mM) purified AD and AD’s site-directed mutant protein were exchanged into a labeling buffer and labeled by the dye NT-647-NHS at room temperature for 30 min in the dark. Then binding affinity was done. The compound was diluted to a maximum concentration of 2mM, then 16 tubes were diluted in 2-fold concentration gradient. Each tube contained 10ul of the diluted solution, and 10uL of the diluted protein solution was added and mixed. The mixture was then pipetted into a capillary and placed in the instrument for detection. (The protein on-machine concentration was 80nM). The assay was performed in triplicates.

### 2.14 Determination of NO and CAT Levels

The MRSE, the MRSE and *ΔarcA* treated with hexadecanal were incubated at 37˚C for 18h. Then the cells were collected by centrifugation (5500rpm, 10min) for use in subsequent assays. The NO and CAT (Liu et al., 2021; Li et al., 2022) in the supernatant were estimated by using kits from Nanjing Jiancheng Institute (Nitrate reductase method). Each treatment was repeated at least three times.

### 2.15 Statistical analysis

Values were expressed as mean ± SEM. For comparisons among three or more experimental groups, one-way analysis of variance (one-way ANOVA) was first conducted. When a significant overall intergroup difference was detected, appropriate post hoc multiple comparison tests (Tukey’s or Bonferroni test) were subsequently applied for pairwise comparisons with correction for multiple comparison bias. Two-sided Student’s t-test was used for comparisons between two independent groups. Values of *p* < 0.05 were considered statistically significant (*p < 0.05; **p < 0.01; ***p < 0.001; ****p < 0.0001). All statistical analyses were performed using GraphPad Prism version 10.0 software (GraphPad, San Diego, California, USA).

## 3 Results

### 3.1 AD as a promising antibacterial target of Patrinia scabiosifolia against MRSE

**Figure 2:**
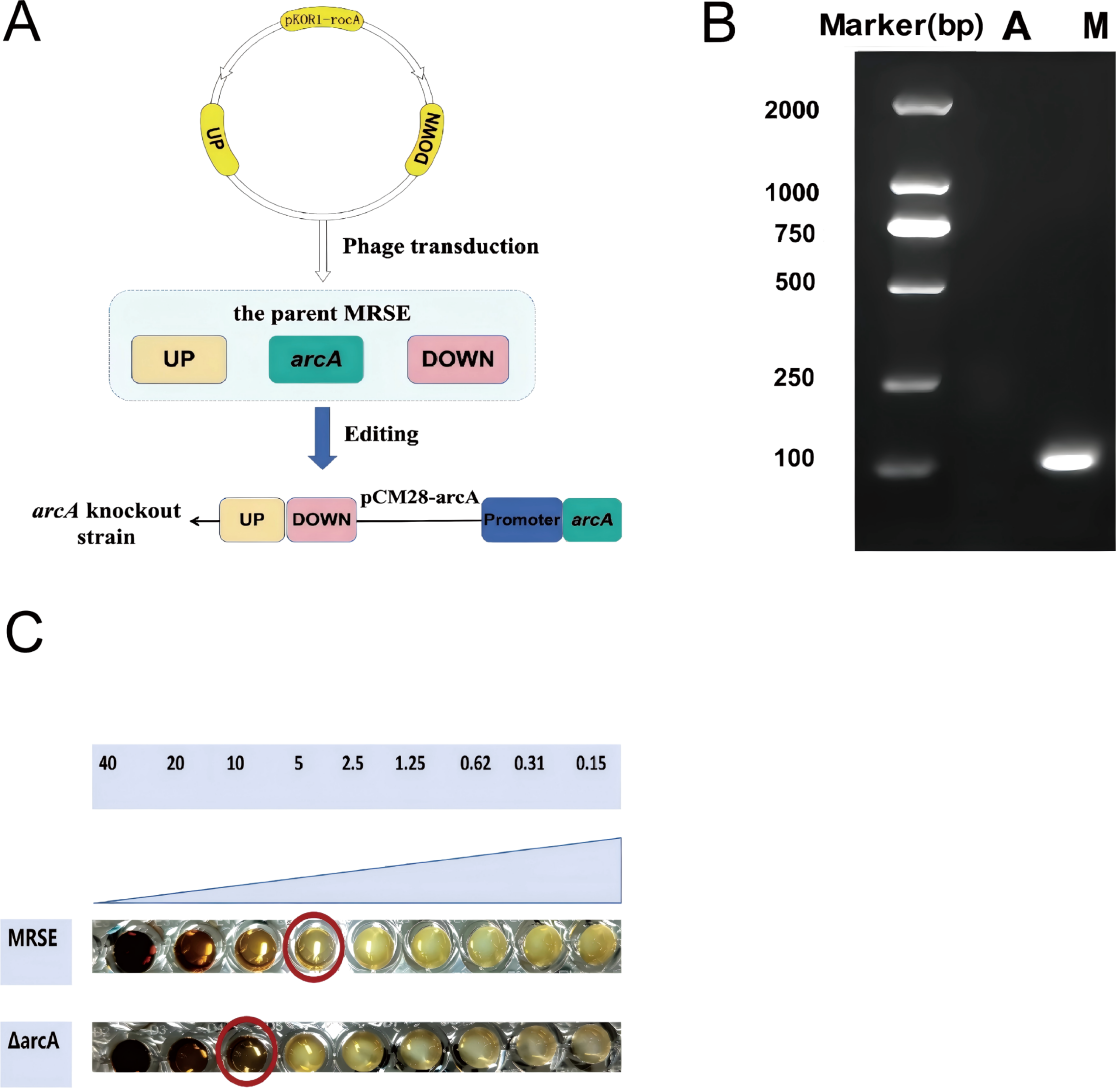
*arcA* as a target for antibacterial action in *Patrinia scabiosifolia.* **(A)** Construction of *arcA* Knockout Strain. Construction of the *arcA*-deficient mutant (*ΔarcA*) via phage transduction in MRSE. A suicide plasmid pKOR1-rocA carrying the upstream (UP) and downstream (DOWN) homologous arms flanking the *arcA* gene was introduced into the parental MRSE strain by phage transduction. Double-crossover homologous recombination was then performed to replace the endogenous *arcA* gene with the UP-DOWN cassette, generating the *arcA* knockout strain. For genetic complementation, the full-length arcA gene with its native promoter was cloned into the shuttle vector pCM28 to construct the complementation plasmid pCM28-*arcA*. **(B)** PCR Verification of *arcA* Gene Deletion. Molecular validation of the *ΔarcA* knockout strain by PCR. Lanes labeled “A” and “M” correspond to the PCR amplification products of the *arcA* gene in the *ΔarcA* mutant and the parental MRSE strain, respectively. The PCR products were separated on a 1.5% agarose gel. Lane 1: DL2000 DNA ladder (2000-100 bp). Lane 2: Product from MRSE, showing the full-length arcA-containing fragment. Lane 3: Product from *ΔarcA*, showing a truncated fragment (~100 bp) corresponding to the deleted locus. The size difference between the two amplicons confirms the successful generation of the *ΔarcA* knockout strain. **(C)** Antibacterial Susceptibility Testing. The graph and images below it show the results of testing the antibacterial susceptibility of two bacterial strains to *Patrinia scabiosifolia* at various concentrations (0.15 mg/mL to 40 mg/mL). The red-circled wells indicate the MIC: 5 mg/mL for the MRSE strain and 10 mg/mL for the *ΔarcA* mutant. The images depict the growth of bacteria in the wells at these MIC concentrations.

Previous proteomic studies revealed that *Patrinia scabiosifolia’s* antimicrobial effect on MRSE triggers AD gene downregulation, suggesting AD as a potential antimicrobial target. To validate this, study employed gene knockout technology to investigate AD’s potential as a therapeutic target. The allelic replacement was used to construct an MRSE *arcA* knockout (*ΔarcA*) (**Figure 2A**), and the outcomes of these processes are validated by PCR (**Figure 2B**). For detailed construction results, please refer to **Supplementary Figure S1**. Using MRSE and *ΔarcA*, the research team conducted a comparative analysis of MIC. **Figure 2C** showed that the MIC of *Patrinia scabiosifolia* against MRSE is 5 mg/mL, but *ΔarcA* is 10 mg/mL. These results demonstrated that AD is a promising antibacterial target of *Patrinia scabiosifolia* against MRSE.

### 3.2 Hexadecanal as bioactive compound of *Patrinia scabiosifolia* against MRSE targeting AD

The results above indicated that AD is a target for *Patrinia scabiosifolia* against MRSE, but the bioactive compound responsible for its antibacterial effects is unknown. Therefore, this study utilizes Computer Virtual Screening Technology to investigate the bioactive compound of *Patrinia scabiosifolia* based on AD.

#### 3.2.1 Computational screening and prioritization of AD-targeting compounds

The three-dimensional structure of *S. epidermidis* AD was generated using AlphaFold2 and validated through standard stereochemical assessments, confirming its suitability for computational analyses. A total of 158 constituents from *Patrinia scabiosifolia* were retrieved from the TCMSP database and filtered using OB ≥ 30% and DL ≥ 0.18 thresholds. Following geometry optimization and protonation-state refinement, all candidates were docked into the predicted AD active site using Glide (SP mode).Virtual screening was initially performed to identify small molecules capable of binding to AD. To avoid bias introduced by docking scores alone, all compounds that successfully docked to the target were considered for experimental validation. Based on structural diversity and commercial availability, 21 compounds were selected for antibacterial activity assays (**Supplementary Table S4**).

#### 3.2.2 Antibacterial activity of the bioactive compound of *Patrinia scabiosifolia* against MRSE *in vitro*

**Figure 3:**
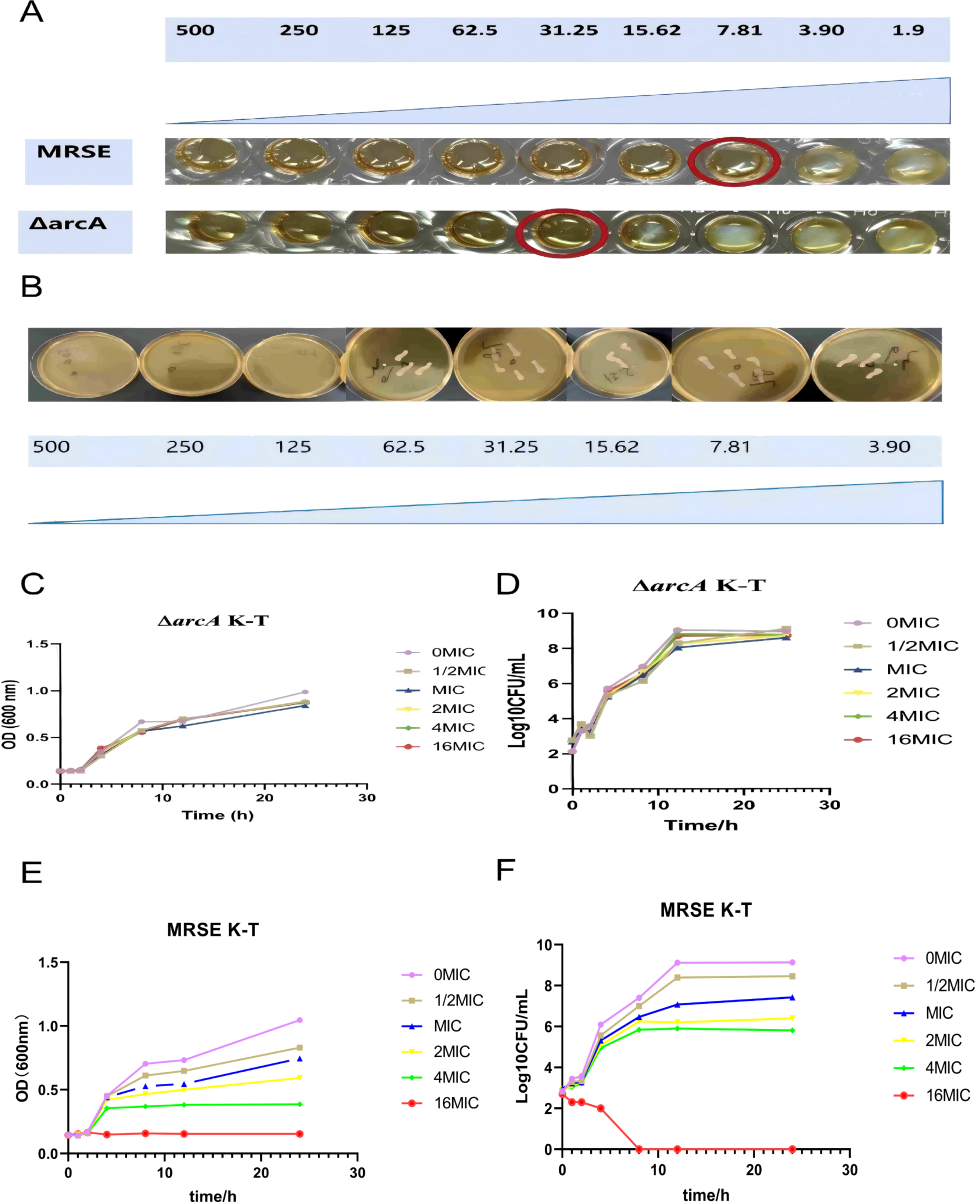
Analysis of hexadecanal as the bioactive compound. **(A)** Minimum Inhibitory Concentration (MIC) of Hexadecanal against *ΔarcA* and MRSE. The image shows a series of petri dishes with varying concentrations of hexadecanal ranging from 500 to 1.9 µg/mL. The MIC is indicated by the lowest concentration that inhibits visible bacterial growth. For MRSE, the MIC is marked by a red circle 7.81 µg/mL, while for *ΔarcA*, it is also circled 31.25 µg/mL. **(B)** Minimum Bactericidal Concentration (MBC) of Hexadecanal against MRSE. This image displays petri dishes with concentrations of hexadecanal from 500 to 3.90 µg/mL. The MBC is determined by the lowest concentration that kills all bacteria, as indicated by the absence of visible colonies.For MRSE, the MBC is 125µg/mL, while for *ΔarcA*, do not appear MBC. **(C)** Growth Curve of *ΔarcA* under Different Concentrations of Hexadecanal. The graph plots the optical density (OD) at 600 nm against time for *ΔarcA* under various concentrations of hexadecanal (0 MIC, 1/2 MIC, MIC, 2 MIC, 4 MIC, 16 MIC). The OD values are used to monitor bacterial growth over time. **(D)** Colony Count of *ΔarcA* under Different Concentrations of Hexadecanal. This graph shows the logarithm of colony-forming units (CFU/mL) over time for *ΔarcA* under different hexadecanal concentrations. The data points are plotted against time to assess the impact of hexadecanal on bacterial colony formation. **(E)** Growth Curve of MRSE under Different Concentrations of Hexadecanal. Similar to panel C, this graph plots the OD at 600 nm against time for MRSE under various hexadecanal concentrations, tracking bacterial growth over time. **(F)** Colony Count of MRSE under Different Concentrations of Hexadecanal. This graph presents the logarithm of CFU/mL over time for MRSE under different hexadecanal concentrations, evaluating the effect of hexadecanal on bacterial colony formation.

A total of 21 candidate small molecules from *Patrinia scabiosifolia*, MIC test of MRSE strains according to CLSI standard showed that hexadecanal was the strongest antibacterial activity (**Supplementary Table S4**). Therefore, hexadecanal can be considered as the most potential active ingredient of *Patrinia scabiosifolia*. In order to verify this view, the research group conducted MIC, MBC and time-kill curves for hexadecanal. The results demonstrated that the MIC of hexadecanal against MRSE was 7.81 μg/mL (**Figure 3A**), and the MBC was 125 μg/mL (**Figure 3B**). In further time-bactericidal curve assays, the bacterial count of MRSE significantly decreased with increasing drug concentrations (**Figures 3E, F**), indicating the antibacterial activity of hexadecanal. Compared to MRSE, the MIC of hexadecanal against *ΔarcA* increased fourfold to 31.25 μg/mL (**Figure 3A**). However, the MBC of hexadecanal against *ΔarcA* could not be quantified at the corresponding concentrations. Additionally, the time-bactericidal curves of hexadecanal against *ΔarcA* showed no significant changes across different concentrations, merely reflecting a trend of bacterial growth over time (**Figures 3C, D**). These results demonstrated that hexadecanal is identified as the potential bioactive compound of *Patrinia scabiosifolia* targeting AD.

#### 3.2.3 Modulate AD to exert hexadecanal’s therapeutic potential for BSI

**Figure 4:**
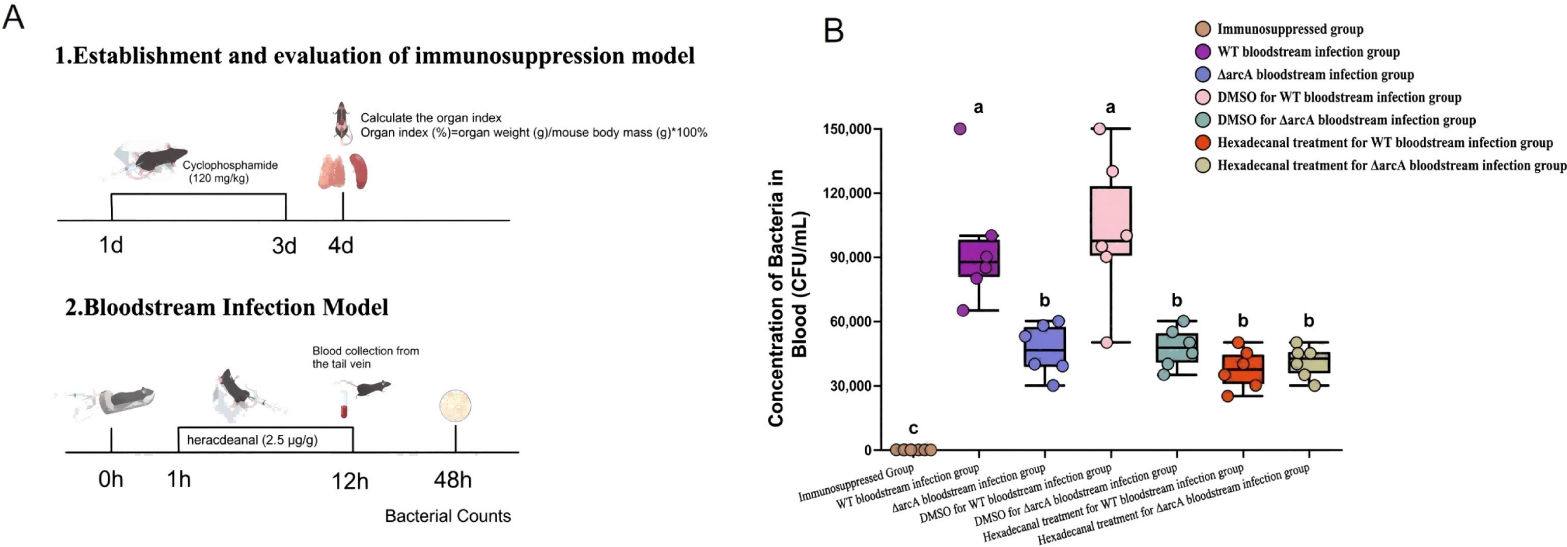
Examination of hexadecanal’s efficacy in addressing bloodstream infections via AD therapy. **(A)** Establishment Pattern Diagram for Bloodstream Infection Models Immunosuppression Model Setup: This diagram details the timeline for establishing the immunosuppression model. Cyclophosphamide (120 mg/kg) is administered on day 1 and 3. Subsequent assessments occur on days 4. The organ index is calculated as follows: Organ index (%) = (organ weight/body weight) × 100 (Lazic et al., 2020). Bloodstream Infection Model: Following cyclophosphamide injection, hexadecanal (2.5 µg/g) is intraperitoneal injection at both the 1h and 12h marks. Bacterial counts are precisely measured at the subsequent 48h interval. **(B)** Validation of Bloodstream Infection Model. The graph presents the concentration of bacterial blood (CFU/mL) across different treatment groups. The data are represented as box plots, with different letters indicating statistical significance among the groups.

Following the acquisition of *in vitro* antimicrobial activity results for hexadecanal, we first established a BSI model to evaluate its *in vivo* antimicrobial efficacy (**Figure 4A**). As shown in **Figure 4B**, both the WT bloodstream infection group and the *ΔarcA* bloodstream infection group exhibited significant differences compared to the immunosuppressed group, indicating successful establishment of the BSI model. Comparison between the WT bloodstream infection group and the DMSO for WT bloodstream infection group revealed no significant difference in bacterial colony counts, suggesting that this concentration of DMSO itself lacks antimicrobial activity. Further comparison between the WT bloodstream infection group and the hexadecanal treatment for WT bloodstream infection group demonstrated a significant reduction in blood colony counts, confirming the pronounced antimicrobial effect of hexadecanal *in vivo*. Similarly, comparison between the *ΔarcA* bloodstream infection group and the DMSO for *ΔarcA* bloodstream infection group also indicated no antimicrobial activity of this DMSO concentration. In contrast, no significant difference in colony counts was observed between the *ΔarcA* bloodstream infection group and the hexadecanal treatment for *ΔarcA* bloodstream infection group, suggesting that the antimicrobial effect of hexadecanal may be arcA-dependent. In conclusion, hexadecanal exhibits therapeutic effects on the WT bloodstream infection model but shows no efficacy against the *ΔarcA* bloodstream infection model, indicating that hexadecanal exerts its antibacterial action by targeting the AD pathway, thereby effectively treating bloodstream infections.

#### 3.2.4 Hexadecanal exerts antibacterial effects against MRSE via modulating AD

**Figure 5:**
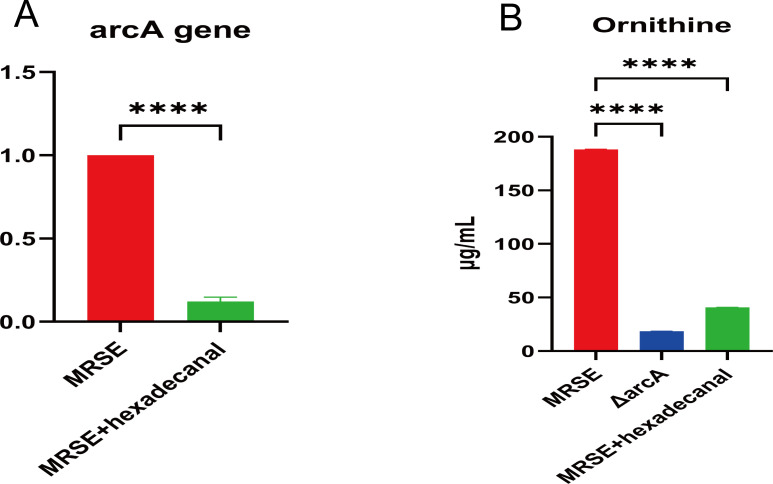
Hexadecanal’s regulatory effect on MRSE expression measurement. **(A)** Hexadecanal significantly reduced *arcA* gene expression in MRSE from approximately 1.0 in controls to 0.1, with high statistical significance (****p < 0.00001). **(B)** Differences in ornithine content under the three treatment conditions: Compared with the MRSE group, the ornithine content in the *ΔarcA* group was significantly lower (****p <0.00001); similarly, the ornithine content in the MRSE group treated with hexadecanal was significantly lower than that in the MRSE group (****p <0.00001).

Based on the finding that AD is the target protein for hexadecanal’s antibacterial activity, we further explored the mechanism of hexadecanal’s action on AD. First, the regulatory effect of hexadecanal on AD was examined, we observed Hexadecanal significantly reduced arcA gene expression in MRSE approximately (**Figure 5A**). In **Figure 5B**, the ornithine concentions in the *ΔarcA* group was significantly reduced compared with the MRSE. Notably, hexadecanal treatment also significantly reduced ornithine levels in MRSE. Collectively, these results suggest that hexadecanal inhibits arcA gene expression, thereby disrupting AD mediated arginine metabolism and reducing ornithine production. 

#### 3.2.5 Molecular Dynamics Simulations Reveal the Structural Basis of Hexadecanal Binding

**Figure 6:**
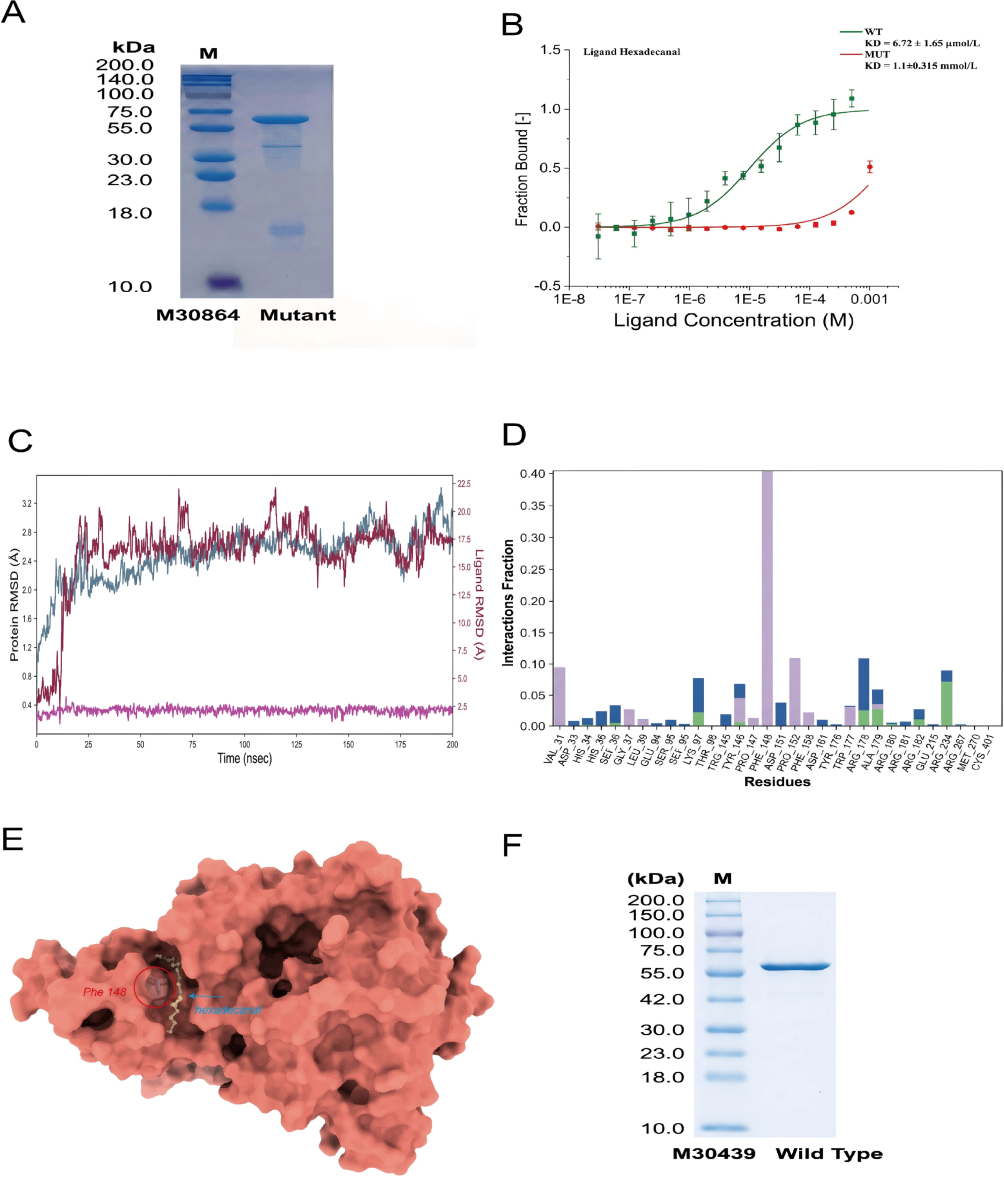
Exploring the binding interaction between hexadecanal and AD **(A)** SDS-PAGE Analysis of AD Expression. The gel image shows the expression levels of AD, with molecular weight markers on the left. The band corresponding to AD is visible at approximately 42 kDa. **(B)** MST Assay: Binding Affinity of Hexadecanal to Wild-Type and Mutant AD. The graph depicts the binding affinity of hexadecanal to AD and its mutant form (MUT) using microscale thermophoresis. The x-axis represents the ligand concentration (M), and the y-axis shows the fraction bound (-). The green line indicates the binding of hexadecanal to WT AD, while the red line shows the binding to the MUT form. The dissociation constant (KD) values are provided for both WT and MUT. **(C)** Molecular dynamics simulation and stability evaluation. The graph presents two datasets plotted against time (nsec): Protein RMSD (Root Mean Square Deviation) in blue and Ligand RMSD in red. The x-axis represents time, ranging from 0 to 200 nsec. The y-axis on the left shows the Protein RMSD values, ranging from approximately 0.4 Å to 3.2 (Å) The y-axis on the right shows the Ligand RMSD values, ranging from approximately 2.5 Å to 22.5 (Å) Both the Protein RMSD and Ligand RMSD exhibit fluctuations over time, indicating dynamic changes in their structures. **(D)** This bar graph illustrates the binding fractions of various amino acids to hexadecanal. The x-axis lists the amino acids, and the y-axis shows the binding fractions. Notably, Phe 148 shows a significant binding fraction. **(E)** This diagram shows hexadecanal binds to Phe148 in the protein cavity, where the small molecule forms a hydrophobic interaction with the Phe148 amino acid. **(F)** SDS-PAGE Analysis of MUT AD Expression: Similar to panel A, this gel image shows the expression levels of the MUT form of AD. The band corresponding to MUT AD is visible at approximately 42 kDa, alongside molecular weight markers.

In addition, the interaction between hexadecanal and AD was examined. The AD protein was expressed (**Figure 6A**) and then MST was used to measure their interaction. The results showed that hexadecanal could interact with AD and the dissociation constant Kd had a value of 6.72 ± 1.65 μmol/L (**Figure 6B**). Meanwhile, we found the interaction intensity was dose-dependent when the concentration of hexadecanal was in the range of 500uM-0.0035 uM.

To elucidate the molecular basis of hexadecanal–AD interactions, 200-ns MD simulations were conducted. The AD–hexadecanal complex demonstrated stable conformational behavior, with backbone RMSD values stabilizing within 2.0–3.0 Å and ligand RMSD within 2.0–3.0 Å (**Figure 6C**). Notably, as shown in **Figure 6D**, Phe148 maintained persistent π–π stacking and hydrophobic interactions with hexadecanal for more than 40% of the simulation time, identifying this residue as a critical binding hotspot. Additional stabilizing interactions were contributed by Val31 and hydrogen bonds involving Arg234. These findings suggested that Phe148 plays a central role in stabilizing hexadecanal binding within the AD active site.

One crucial binding site (Phe148) was accurately predicted (**Figure 6E**), and corresponding site-directed mutant proteins were successfully constructed (**Figure 6F**). We further probed the interaction between hexadecanal and the mutated protein using MST. **Figure 6B** reveals that the mutant protein’s dissociation constant (Kd) plummeted by over 100-fold to a value of 1.1 ± 0.315 mmol/L. In summary, these compelling results strongly indicate that hexadecanal binds to AD specifically through Phe148.

#### 3.2.6 Determine of nitric oxide (NO) and catalase (CAT)

**Figure 7:**
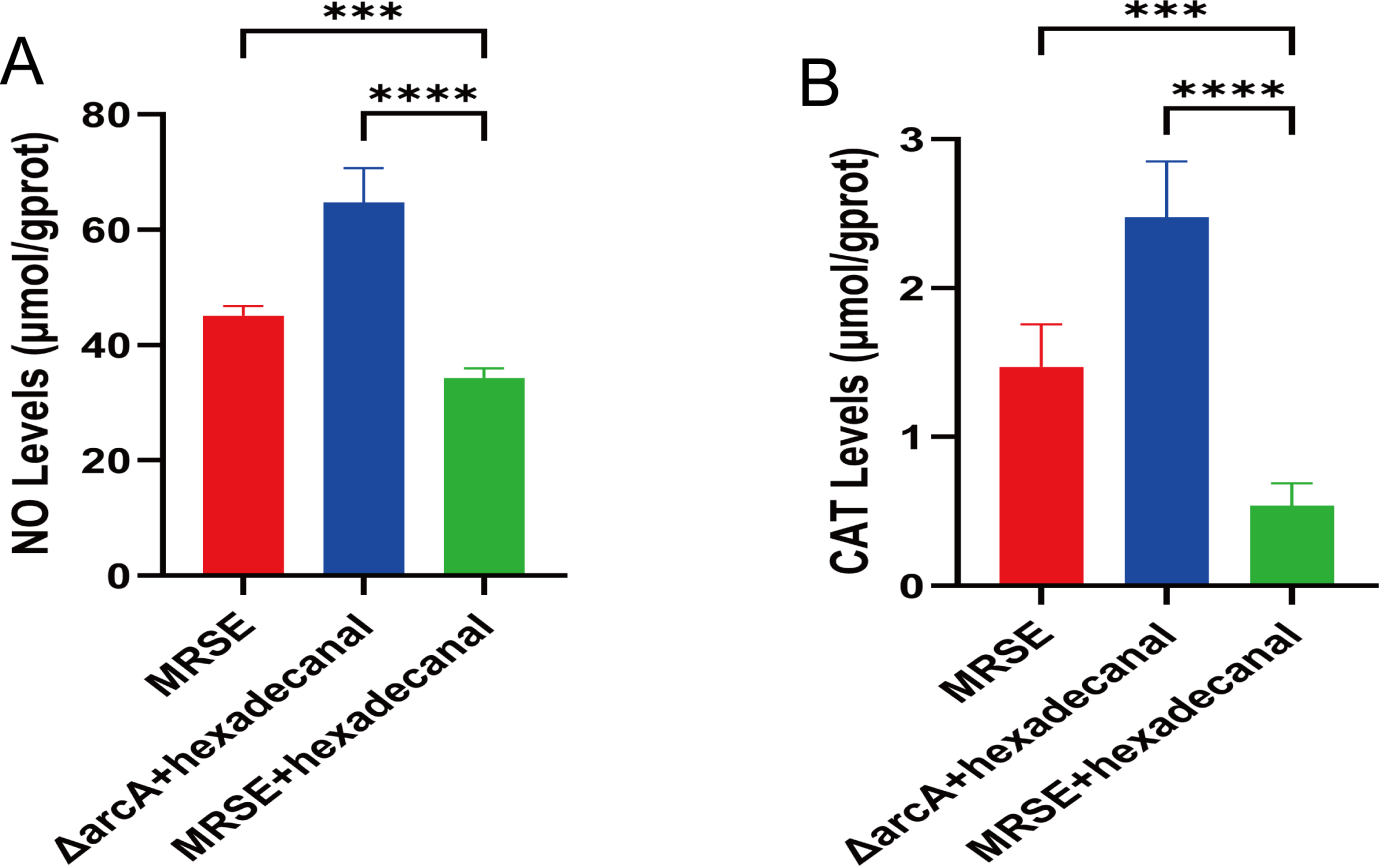
Analysis of CAT and NO levels in bacteria. **(A)** NO levels. The bar graph compares NO levels across three experimental groups: MRSE, *ΔarcA* treated with hexadecanal and MRSE treated with hexadecanal. Compared to MRSE, NO levels were significantly suppressed under hexadecanal stress of MRSE (***p < 0.001). Compared to the MRSE treated with hexadecanal, while *ΔarcA* treated with hexadecanal shows markedly rise NO levels (****p < 0.00001). **(B)** CAT levels. The same three groups as those in **Figure 7A**, Compared to MRSE, CAT levels were significantly suppressed under hexadecanal stress of MRSE (***p < 0.001). Compared to the MRSE treated with hexadecanal, while *ΔarcA* treated with hexadecanal shows markedly rise CAT levels (**** *p* < 0.00001).

**Figures 7A, B** demonstrate that compared with the MRSE group, NO and CAT levels decreased after the MRSE treated with hexadecanal (green), suggesting that hexadecanal exerts its antibacterial effect by inhibiting NO and CAT levels. However, no significant reductions in NO and CAT levels were observed in the *ΔarcA* treated with hexadecanal following hexadecanal treatment, indicating that hexadecanal fails to downregulate NO and CAT to exert antibacterial levels upon the deletion of its target AD.

## 4 Discussion

With the widespread use of invasive diagnostic and therapeutic techniques, there is a trend of increasing incidence of bloodstream infections. Meanwhile, the WHO reported that prolonged catheter indwelling is associated with an increased risk of bloodstream infections, which may elevate the mortality rate among patients with COVID-19 during the establishment of deep venous access in critically ill patients diagnosed with COVID-19 (Udoakang et al., 2023). According to recent national surveillance data on bloodstream infections, the isolation rate of *coagulase-negative staphylococci* is between 19-35% and ranks among the top ten pathogens causing BSI. Of these, *Staphylococcus epidermidis* are the most common (Rather et al., 2023). Seriously, the rate of MRSE isolation is as high as 88.4% (Laura et al., 2020; Catteau et al., 2023). Furthermore, all currently available drugs for treating MRSE infections have severe adverse effects, and medium-resistant strains have already emerged. Therefore, the search for novel antibacterial agents that can inhibit and kill MRSE remains urgent.

Previous studies in our research group have shown that Patrinia scabiosifolia antibacterial activity against MRSE and AD may be the target protein for its antibacterial action (Liu et al., 2023). Therefore, arcA was knocked out to support the above inference, and the results showed that the MIC of the ΔarcA was twice as high as that of the MRSE (**Figure 2C**). As one of the predominant arginine metabolic pathways, the arginine deiminase (Sullivan et al., 2021) pathway supplies bacteria with citrulline, ATP and other essential substrates required for proliferation. Meanwhile, this pathway enhances bacterial tolerance against acid stress, salt stress and antibacterial agents (Lin et al., 2020). In Staphylococcus aureus, the arginine deiminase pathway contributes to the development of vancomycin resistance in bacteria (Tan et al., 2017). Furthermore, accumulating evidence has revealed differential arcA expression in drug-resistant strains of Enterococcus (Todd Rose et al., 2023) and *Streptococcus* (Rued et al., 2022), suggesting a close correlation between this gene and bacterial drug resistance. Our results also demonstrated that AD was one of the targert protein of *Patrinia scabiosifolia* against MRSE.

In order to clarify the relationship between the pharmacological effects and chemical components, the research on the bioactive compound of TCM is an inevitable requirement for the modernization of TCM by modern science to elucidate the safety and efficacy. Thus this study investigates the bioactive compound in *Patrinia scabiosifolia*. Currently, there are several methods used to study the bioactive compound of TCM, such as the study of effective components, pharmaco-spectrum relationships, serum pharmacology, metabolomics, computer-assisted drug design (CADD) and so on. In contrast to conventional high-throughput screening, CADD is grounded in the principles of structural biology, significantly reducing both research costs and time while mitigating the challenges of randomness and limitations inherent in the high-throughput screening process (Amisha et al., 2024). And it is widely used in reverse drug targeting for bioactive compound of TCM and virtual screening of bioactive compound in TCM (Wang et al., 2022; Tan et al., 2023). Consequently CADD was used to virtual screening bioactive compound in *Patrinia scabiosifolia* by targeting AD. This study employed molecular docking and molecular dynamics (MD) simulation techniques to screen the bioactive compound in *Patrinia scabiosifolia* with MRSE activity.

Among the 21 screened bioactive compounds, hexadecanal exhibited the most significant antibacterial properties. Vancomycin was also selected as the control, with a MIC of 1.9 μg/mL(**Supplementary Table S5**). Although the MIC of vancomycin was lower than that of hexadecanal (7.81 μg/mL), although vancomycin is universally recognized as a specific standard antibiotic against *Staphylococcus aureus*, we conducted further studies on hexadecanal to elucidate its therapeutic efficacy against MRSE, with the hope that this compound may serve as a potential alternative therapy for MRSE in the future. The hexadecanal is also known as palmitaldehyde, which was essential oils (EOs) (Yang et al., 2023). EOs are natural secondary metabolites derived from plants, recognized for their utility in food and nutraceutical production, perfumery, and cosmetics. Additionally, they have been shown to mitigate the issue of antimicrobial resistance (Amisha et al., 2024). while also serving as effective antibacterial agents (Aljaafari et al., 2022). Among these, cinnamaldehyde had a great role in antimicrobial activity, including *E.coli, Salmonella typhimurium, Staphylococcus aureus*, and *Vibrio parahaemolyticus* (Doyle and Stephens, 2019; Hassan et al., 2024). At the same time, it also shows good effects in inhibiting bacterial biofilm formation (Didehdar et al., 2022). However, there has been no research on the antibacterial and mechanism of hexadecanal at present. Thus, we conducted further studies on hexadecanal to elucidate its therapeutic efficacy against MRSE, with the hope that this compound may serve as a potential alternative therapy for MRSE in the future. The antibacterial efficacy of hexadecanal against MRSE and *ΔarcA* in vitro and in vivo were rigorously evaluated, the results revealed that in comparison to MRSE, the antibacterial activity of hexadecanal against the *ΔarcA* decreased by fourfold in vitro, and its therapeutic efficacy against bloodstream infection induced by the *ΔarcA* was markedly reduced. This suggests that hexadecanal may exert antibacterial effects through AD. Accordingly, further investigations were performed to elucidate the antibacterial mechanism of hexadecanal targeting AD. Firstly, the regulatory role of hexadecanal on AD is explored by measuring the effects of hexadecanal on *arcA* expression and the ornithine levels. The results showed that hexadecanal inhibited *arcA* expression (**Figure 5A**). Meanwhile, compared with MRSE, ornithine content was markedly reduced in the *ΔarcA*, demonstrating that arcA is the key enzyme catalyzing arginine conversion into ornithine in MRSE. Moreover, hexadecanal significantly lowered ornithine levels in MRSE, indicating that hexadecanal reduces ornithine production by suppressing arcA expression. On the other hand, researchers investigated the binding of hexadecanal with AD using MST to determine whether hexadecanal could bind to AD. MD method predicted that hexadecanal has binding capability with the AD crystal structure, with its binding site being phe148. To validate the binding capability, after mutating the amino acid at the binding site phe148 and expressing the mutant, MST was previously reported was employed again to verify the binding site of hexadecanal. Also, MST once again proved that the binding site of AD with hexadecanal was Phe 148. It was ultimately confirmed that hexadecanal from *Patrinia scabiosifolia* is an effective antibacterial component targeting AD.

Nitric oxide (NO) (Rios-Soto et al., 2024), an inorganic signaling molecule endogenously synthesized by both prokaryotes and eukaryotes, is produced from arginine via the catalytic reaction of bacterial NO synthase (bNOS) (Song et al., 2021; Rather et al., 2023). Intracellular NO is closely interlinked with the oxidative stress response of staphylococcal pathogens including MRSE (Singh et al., 2023). Given the above background, we further explored whether the anti-MRSE of hexadecanal is linked to NO-mediated pathways. As shown in **Figure 7**, hexadecanal treatment markedly reduced intracellular NO and CAT levels in MRSE. This indicates that hexadecanal exert their antibacterial effects by reducing the levels of NO and CAT. However, hexadecanal failed to significantly downregulate NO and CAT levels in the *ΔarcA*, indicating that upon *arcA* deletion, hexadecanal loses the capacity to modulate NO and CAT to produce effective antibacterial activity, further verifying that *arcA* serves as the molecular target for hexadecanal’s antibacterial action. Notably, NO and CAT levels were significantly elevated in the *ΔarcA* after hexadecanal treatment. This is because permanent loss of *arcA* in the knockout strain disrupts the arginine deiminase pathway and causes massive arginine accumulation. To compensate chronically at the metabolic level, bacteria upregulate bNOS and antioxidant systems, which ultimately results in markedly higher basal NO and CAT levels in the knockout mutant compared with the wild-type strain.

## 5 Conclusions

Hexadecanal is the bioactive constituent of Patrinia scabiosaefolia responsible for its antibacterial effect via targeting AD. By binding to and modulating *arcA*, hexadecanal reduces intracellular NO levels, suppresses bacterial antioxidant responses and ultimately exerts antibacterial activity.

## Data Availability

The original contributions presented in the study are included in the article/**Supplementary Material**. Further inquiries can be directed to the corresponding author.
