# Supplementary material for: Active components of Patrinia scabiosifolia and mechanisms of action against methicillin-resistant Staphylococcus Epidermidis
Source: Front Cell Infect Microbiol. 2026 Jul 15;16:1813767. doi: 10.3389/fcimb.2026.1813767 (PMC13414910; doi:10.3389/fcimb.2026.1813767)
Supplement: Supplementary file 1 [file Table1.docx]

Active Components of *Patrinia scabiosifolia* and Mechanisms of Action Against Methicillin-Resistant *Staphylococcus Epidermidis*

Qiantonghan Luo^1+^, Wenqiang Cui^2+^ ,Meng Ni^3^，Yuqi Yang^1^,Yonghui Zhou^1^，Lili An^3^ and Xin Liu^1^ *

^1^Guizhou University of Traditional Chinese Medicine, Guiyang City, People’s Republic of China

^2^The Research Center for Computer-aided Drug Discovery, Institute of Biomedicine and Biotechnology, The Shenzhen Institute of Advanced Technology, Chinese Academy of Sciences, Shenzhen 518055, China

University of Chinese Academy of Sciences, Beijing 100049, China

^3^The First Affiliated Hospital of Guizhou University of Traditional Chinese Medicine, Guiyang City, People’s Republic of China

^+^These authors contributed equally to this work and share first authorship

*** Correspondence:**Xin Liu

1046977102@qq.com

Keywords: Methicillin-resistan *Staphylococcus epidermidis*; Bloodstream infections; *Patrinia scabiosifolia*; Arginine deiminase; Hexadecanal

# Supplementary Figures and Tables

**
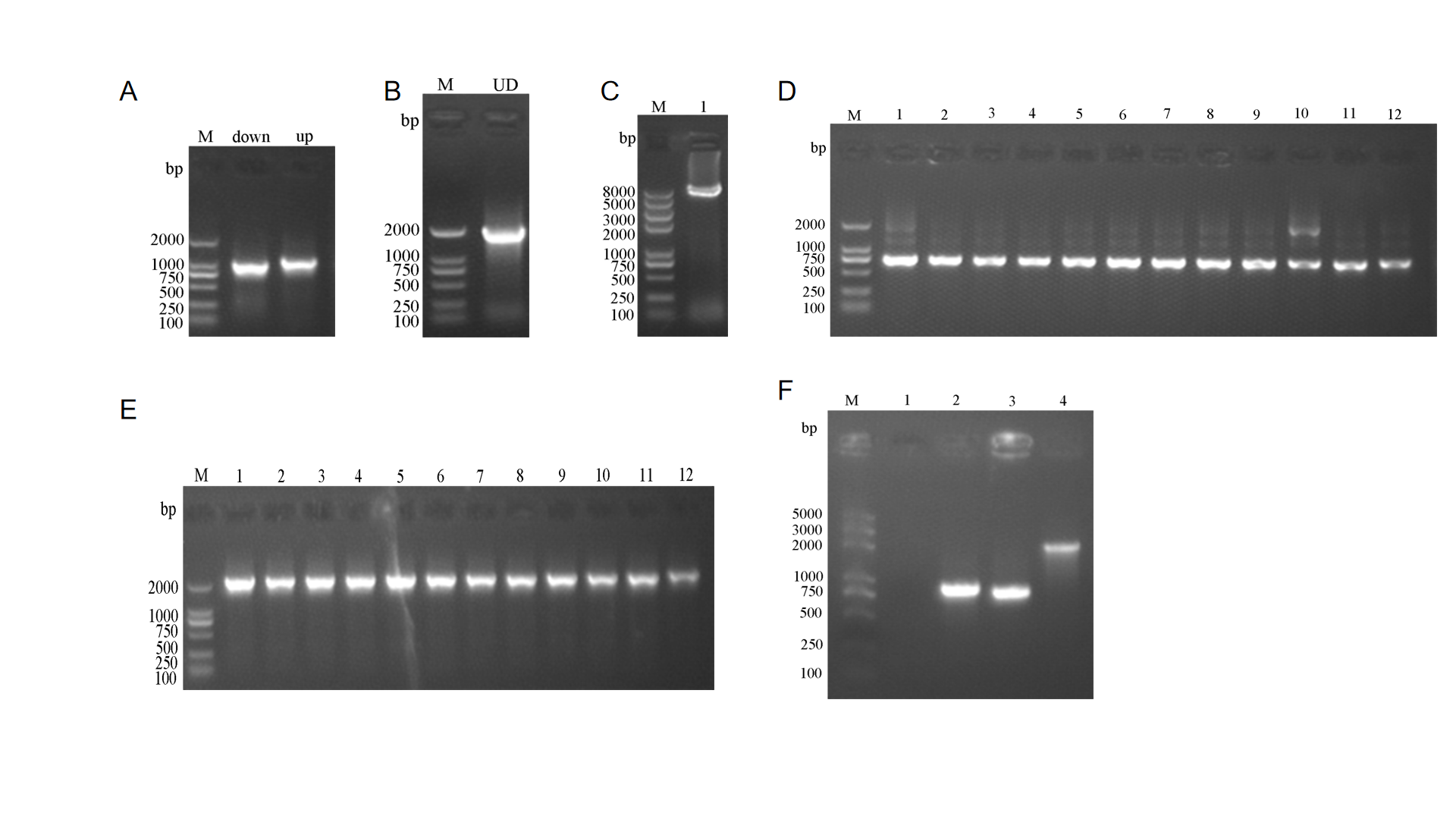
Figure 1S The process of constructing MRSE *arcA* gene deletion strain and verification**

1. *arcA* upstream and downstream homologous arm amplification products were subjected to detection using 1% agarose gel electrophoresis.
2. *arcA*-pKOR1 amplification agarose gel electrophoresis detection
3. pKOR1 amplification by agarose gel electrophoresis
4. Cytogenetic vector amplification by agarose gel electrophoresis: 1-12 shows randomly selected colony clones
5. Cocktail vector transformation ATCC12228 colony PCR identification
6. *arcA*-JD-F、*arcA*-JD-R、*arcA*-ter-F and *arcA*-ter-R identify wild bacteria and knocked out bacteria: 1 shows the amplification products of the knockout strain ATCC12228△ *arcA*-ter-F; 2 shows the amplification products of the ATCC12228 strain *arcA*-ter-R; 3 shows the amplification products of the ATCC12228△*arcA* strain *arcA*-JD-F and 4 shows the amplification products of the wild-type ATCC12228 strain arcA-JD-R.

| **Table 1S Elution Gradient** | | |
| --- | --- | --- |
| **Time( min)** | **A%** | **B%** |
| 0.01 | 5 | 95 |
| 4 | 20 | 80 |
| 6 | 20 | 80 |
| 12 | 45 | 55 |
| 12.1 | 5 | 95 |
| 17 | Stop |  |

Table 1S The table details the time-dependent proportions of mobile phase A (water, pH 3) and mobile phase B (90% acetonitrile-10% water, pH 3) used in the HPLC analysis of ornithine in MRSE samples treated with or without hexadecanal.

| **Table 2S Source Parameters** | | |
| --- | --- | --- |
| **Parameter** | **Value (+)** | **Value (-)** |
| Gas Temp (°C) | 350 | 350 |
| Gas Flow (l/min) | 10 | 10 |
| Nebulizer (psi) | 45 | 45 |
| Capillary (V) | 4000 | 3500 |

Table 2S Mass spectrometry source parameters.Electrospray ionization (ESI) source parameters (positive and negative ion modes) used for the detection of ornithine and related metabolites.

| **Table 3S Scan Segments** | | | | | | | |
| --- | --- | --- | --- | --- | --- | --- | --- |
| **Cpd Name** | **Prec Ion MS1 Res** | **Prod Ion MS2 Res** | **Dwell** | **Frag (V)** | **CE (V)** | **Cell Acc (V)** | **Polarity** |
| 26-Citrulline | 176. 1 Unit/Enh  (6490) | 159 Unit/Enh  (6490) | 10 | 80 | 6 | 4 | Positive |
| 26-Citrulline | 176. 1 Unit/Enh  (6490) | 70. 1 Unit/Enh  (6490) | 10 | 80 | 24 | 4 | Positive |
| 27-Ornithine | 133. 1 Unit/Enh  (6490) | 116 Unit/Enh  (6490) | 10 | 70 | 6 | 4 | Positive |
| 27-Ornithine | 133. 1 Unit/Enh  (6490) | 70. 1 Unit/Enh  (6490) | 10 | 70 | 18 | 4 | Positive |

Table 3S The table lists the optimized precursor/product ion pairs, dwell time, fragmentor voltage (Frag), collision energy (CE), cell accelerator voltage (Cell Acc), and polarity used for the quantitative detection of ornithine and citrulline in MRSE samples treated with or without hexadecanal.

| **Table 4S MICs of 21 compounds** | | |
| --- | --- | --- |
| **NAME** | **CAS** | **MIC** |
| Gallic acid.sdf | T0877(149-91-7) | Fullymixed |
| 3-Methylbutanoic acid.sdf | T5609(503-74-2) | 1000mg/mL |
| dodecanoic acid.sdf | T6873(143-07-7) | 15.625mg/mL |
| Eucalyptole.sdf | T1714(470-82-6) | 500mg/mL |
| Octadecanal.sdf | T19493(638-66-4) | 500mg/mL |
| Hexadecanal.sdf | T13719(629-80-1) | 7.81μg/mL |
| Farnesol.sdf | T7868(4602-84-0) | 125mg/mL |
| Nerolidol.sdf | T2S2172(7212-44-4) | 1000mg/mL |
| Tetradecane.sdf | IN6959(629-59-4) | 1000mg/mL |
| Protocatechuic acid.sdf | T0562(99-50-3) | 1000mg/mL |
| 1benzofuran sdf | T3234(17092-92-1) | 1000mg/mL |
| Ferulic acid.sdf | T2215(1135-24-6) | 1000mg/mL |
| a-Terpineol.sdf | Fr14115(98-55-5) | 1000mg/mL |
| Nonanoic acid.sdf | T3947(112-05-0) | 500mg/mL |
| Linalool.sdf | T2S2264(78-70-6) | 1000mg/mL |
| Phytol sdf | T3254(150-86-7) | 1000mg/mL |
| Linoleic acid.sdf | T4P2931(60-33-3) | 7.8125mg/mL |
| Hexadecanoic acid.sdf | T2908(57-10-3) | 1000mg/mL |
| Oleic acid.sdf | 2O2668(112-80-1) | Fully mixed |
| Methyl hexadecanoate sdf | T2S0157(112-39-0) | 1000mg/mL |
| Octadecane sdf | T7996(593-45-3) | 1000mg/mL |

Table 4S MICs of 21 *Patrinia scabiosifolia* derived compounds against MRSE.

The table lists compound names, CAS numbers, and corresponding MIC values against MRSE, identifying hexadecanal as the most potent active constituent.

| **Table 5S Comparison chart of the MIC of hexadecanal and vancomycin against MRSE** | | |
| --- | --- | --- |
| **NAME** | **CAS** | **MIC** |
| Hexadecanal.sdf | T13719(629-80-1) | 7.81μg/mL |
| Vancomycin | No.1404-90-6 | 1.9μg/mL |

Table 5S The table lists the MIC values of hexadecanal and the positive control vancomycin against MRSE, determined according to CLSI standards.
